# Collectanea Medica, Consisting of Anecdotes, Facts, Extracts, Illustrations, Queries, Suggestions, &c.

**Published:** 1818-02

**Authors:** 


### COLLECTANEA MEDICA,

CONSISTING OF

### ANECDOTES, FACTS, EXTRACTS, ILLUSTRATIONS, QUERIES, SUGGESTIONS, &c.

RELATING TO THE

*History or the Art of Medicine, and the Auxiliary Sciences.*

Quicquid agunt medici, nostri farrago libelli.

### *The Muscæ Volitantes of Nervous Persons.* By James Ware, Esq. F.R.S.

NO imperfection of vision is more common than that which is occasioned by the appearance of dark-coloured moats before the eyes. These assume various shapes and figures, appear at different distances, and move in different directions, but have no tangible existence in the places where they are seen. Though they do not hinder a distinct perception of the smallest objects, the sight is often much incommoded by them; and the mind is agitated by the apprehension that they are certain precursors of the loss of sight. In the following paper, I propose to give an account of this affection, as it appeared in three different persons; after which I shall offer a few remarks on their proximate cause, their probable termination, and the means that have been employed to accomplish their removal.

Case I.—Mrs. L. was first molested by these appearances in the year 1786, being then about thirty years of age. When she awoke in the morning, some minutes elapsed before she could see any thing distinctly; and then a great number of small yellow moats appeared to dance before every object to which she directed her eyes. At the time I was consulted, the moats had increased considerably in size, though their magnitude depended much on the distance at which they were observed,—being larger when seen far off, and smaller when near the eyes. Besides these yellow sparks, if the eyes were exposed to a strong light, the air appeared full of small particles resembling globules of quicksilver in continual motion; among which were three or four darker coloured than the rest, which appeared as large as small peas, and seemed to turn round on their axes. When the eyes were exposed to a strong light, she perceived several long irregular figures, some of which seemed to be composed of these globules twisted together, and others were like the flue that is swept from bed-rooms. These figures appeared to rise when she lifted her eyes up, and to fall when she turned them down, and they never continued long in one position. If she looked at the sky for two or three minutes, and then turned from it, her eyes flashed fire for the same space of time; and, if she looked at the sun, though but for a moment, she seemed to see four or five suns, which appeared at first like balls of fire, but soon turned green; and still continued visible, though she shut her eyes, and applied her hands before them. All the impressions which a strong light made on the eyes continued for some time after the light was excluded, except the dark-coloured specks, one only of which was perceived when the eyelids were closed; and this also disappeared, if the hand was placed before it.

When the preceding description was given me, both eyes were supposed to be affected in the same way; but, on a closer inquiry, it was found that the right eye only perceived the moats, the left eye being scarcely affected by them.

This lady had been much afflicted, shortly before I saw her, by the death of her husband; her legs and arms trembled, and her heart beat violently. She was much troubled with flatulency, and had a dull heavy pain in the back of her head; which pain sometimes extended to the forehead and sockets of the eyes. She had a strong beating in her temples, with a noise in her ears; and was in general remarkably irritable. Her bowels were in good order, and all the usual evacuations regular as to time and quantity.

On examining the eyes, the pupils appeared perfectly clear; they dilated and contracted freely, on every diminution or increase of light, and the patient distinguished with facility, and with either eye, the smallest characters. Under these circumstances, I did not hesitate to deliver my opinion, that there was not any danger to the sight in the symptom that had so much alarmed her; and my advice was to aim at strengthening the constitution, and particularly the nervous system, by giving two or three times in the day small doses of the volatile tincture of valerian, mixed with an equal quantity of tincture of castor, and joined occasionally with the camphor mixture or infusion of cascarilla. I directed warm water, or a warm infusion of rosemary, to be applied to the eyes and forehead, whenever they felt heavy and uncomfortable; and that the forehead, temples, and outside of the eyelids, should be embrocated morning and evening with a camphorated rosemary spirit. I also recommended a change of residence, with such occupations and amusements as were most likely, without occasioning fatigue, to withdraw the mind from the reflections which had been the source of anxiety and distress. This advice was pursued for a considerable length of time; the moats soon becoming less troublesome, and the mind being relieved from the dread of blindness, which had previously occasioned extreme anxiety.

In the year 1813, that is, twenty-five years after I had been first consulted in this case, I again saw the patient. She then enjoyed good health and spirits. The moats were still occasionally perceived; but they had become so faint, that she could only see them in a strong light, and when she took pains to look for them. It ought, however, to be mentioned, that at this time her daughter was just married; whereas, when she first consulted me, she had lately lost her husband.

Case II.—In the year 1801, I was consulted by the brother of a nobleman, about thirty-one years of age, on account, as he described it, of a considerable number of intersecting moats or beams which floated continually before both his eyes, but particularly before the right. Sometimes they appeared nearly spherical, sometimes like long knotted lines, and sometimes like a series of spherical knots, varying in number, magnitude, and opacity. Sometimes they seemed to have an undulatory motion, and one of them in the right eye was always larger than the rest. They were perceived when the eyelids were closed, almost as strongly as when they were open. When the eyes were directed to an object beyond the usual distance of distinct vision, this object appeared as if it was seen through a pane of glass sprinkled with water. The right eye had been less vigorous than the left for ten years. He had never suffered from head-ach or pain in the eyes; but, when the present morbid sensations began, he had had a violent heat in them, without inflammation, and the heat was followed by a great languor. It had been suspected at one time that an insect had insinuated itself under the right upper eyelid, and much pains were taken to extract it, but of course without success. He had lived three years in a tropical climate, had made free use of mercury, had had much mental agitation, and frequent feverish dreams. He had never been intemperate, and was usually abstemious in his diet. His temper was naturally even; his spirits vigorous; and he had very rarely been subject to hypochondriacism or melancholy. On examining the eyes, they appeared perfect; the pupils were clear, and of their proper size; and they dilated and contracted regularly in different degrees of light. He was near-sighted, though this did not appear by any unusual convexity of the cornea, and the near-sightedness was obviated by the use of a concave glass.

After a careful consideration of the preceding history, I assured the gentleman that his sight was not endangered by any of the symptoms that he described; and the advice I gave was very similar to that which had been given in the preceding case, viz.—that he should endeavour to strengthen the constitution, particularly the nervous system, and abstain as much as possible from every thing likely to agitate the spirits.

Twelve years afterwards, I had occasion to see this gentleman again, when he informed me that he retained the perfect sight of both eyes, and could distinguish the most minute objects with either of them. In a bright light, however, he still perceived the moats as before, if he took pains to look for them; but he was now so much accustomed to their appearance, that they did not occasion any uneasiness. He continued near-sighted, and made use of a concave glass, denominated No. 4, to distinguish objects at a distance.

Case III.—About ten years ago, a lady consulted me, in great distress of mind, on account of the recent appearance of several dark-coloured moats before her eyes. These had no external existence, but assumed fixed figures: that before the right eye was nearly the eighth of an inch wide, and three inches long; its sides were bounded by wavy lines, and its length was interrupted by many apparent knots, being bent near its middle so as to form an obtuse angle: whilst that before the left eye was quite straight, of the same width with the other, about two inches long, and was continued obliquely downward from the right to the left. These appearances were most plainly perceived when she went from one bright light to another, the intermediate space being dark; as, for instance, when she removed from looking through one window in a room to look through another. When the moats were first noticed, they were only seen in a bright light; but after a short time they were also perceived in the shade, becoming, however, much plainer and more numerous when the light was strong. When the lids were shut, a great number of dark-coloured spots were seen before both eyes, but they were not united to form lines until the lids were opened. She had not had pain in the eyes, but her head felt heavy, and was often giddy. Her spirits were easily agitated, and she had continually a rumbling noise in her ears. When she looked from a room into the street, objects appeared larger than natural, and she was obliged to contract her eyelids to bring them to their proper size, distant objects always appearing hazy and confused. The eyes were free from inflammation, and the pupils perfectly clear, dilating and contracting with great readiness in different degrees of light. On looking through a concave glass, the undue magnitude and haziness of distant objects immediately ceased, and she beheld them of their natural size and clearness. The imaginary knots and lines continued to molest her, notwithstanding the use of the glass, but they were not then so strongly marked. On considering all these circumstances, I thought myself justified in assuring the patient that there was not any danger in the symptoms she had described; and, after giving her a medicine to clear the bowels, I advised her to take a drachm of the volatile tincture of valerian, in a cupful of the camphor mixture, two or three times in the day, and to bathe her temples and forehead, and the outsides of her eyelids, morning and evening, with a camphorated rosemary spirit. After this time I did not hear from her professionally during many years, but was occasionally informed that the moats had not wholly disappeared, though they were become so faint, and so little interfered with vision, that she, in a great degree, had lost her anxiety about them. Two years ago I was again consulted on account of a recent discovery that she was unable to read with the left eye: on examining it, I found the pupil perfectly clear, and retaining its full power of dilating and contracting; but I took notice that, when an attempt was made to read, the book was held at a considerable and unusual distance. This was the more remarkable, as the eye had previously been near-sighted. It was now, however, evidently become presbyopic, and, on holding a convex glass of thirty-six inches focus before it, the confusion was removed, and the smallest characters were read without difficulty. Notwithstanding this sudden change in the sight of the left eye, the right eye continued myopic, and required a concave glass to enable it to see distant objects. I again assured the patient that there was not the smallest danger in this new symptom; but, as she was far advanced in pregnancy and naturally of a full habit, I advised that she should lose ten ounces of blood from her arm, and that she should take a few cooling medicines, joining with them some volatile drops, whenever her spirits were much depressed. By this treatment the presbyopic sight of the left eye was in a short time removed; and I have just heard from her family physician that the left eye has now no other defect than that which arises from the muscæ volitantes, which are only occasionally seen, and do not injure vision, and the right eye is rather less near-sighted than it was when I was last consulted.

The three cases of muscæ volitantes, which have been now described, may be considered as examples of a considerable number of the same kind, which, I do not doubt, have fallen under the observation of many gentlemen in this society. It is not easy to ascertain the proximate cause of these moats; but, from the constancy in their figure and their frequently long continuance, it seems probable that they depend on a steady pressure on one or more minute points of the retina, which are situated near the axis of vision, but not exactly in it. The pressure must be near this axis, because the moats always appear near the objects that are looked at; but it cannot be in the axis, because the moats do not injure or impair their natural appearance. As the pressure is not in the axis, the outline of the moats is always somewhat obscure; and the exertion that is made to bring the moats into the axis by moving the eye, gives them an apparent motion, which is sometimes upward and downward, and sometimes from side to side.—*Lond. Med. Soc. Trans.*

---

### *Ophthalmic Form of Fever*; by Robert Jackson, M.D.

Ophthalmia, or rather an ophthalmic form of fever, has been frequent in the British army since the year 1801. It has been more common, perhaps, among those regiments which served the great campaign in Egypt in the year 1800, than among the other corps of the army; but, though this be true, it would be rash to say positively that ophthalmia, as now known, derives from that origin, and is still supported by infections originally imported from that source. The ophthalmic form of fever appears to be sometimes the product of locality;—I cannot pretend, from my own observation, to form an opinion concerning the degree and extent of its infectiousness. A disease which made its appearance under the ophthalmic form, in a troop of hussars of the corps Hompesch, quartered in a sugar plantation in the plain Cul de Sac, in the district of Port au Prince and island of St. Domingo, in the month of December 1796, is strongly corroborative of the opinion of its occasional locality. The indisposition alluded to prevailed so generally in this particular troop, that few escaped from an attack of it. It, in fact, appeared to absorb every other disease; and, when the subjects of it were brought to the hospital which was established at Croix des Bouquets, a village in the neighbourhood, they often experienced a change in the mode of action, viz.—from ophthalmic to dysenteric, and sometimes to intermittent or remittent, more especially where astringent or repellent applications had been made to the eye without previous and copious evacuation. The action of the disease was, in this case, more directed to the appendages than to the ball of the eye; that is, the inflammation was more humoural, rather than dry and ardent.—It was this occurrence in the Hompesch Hussars which first gave me the idea of introducing the ophthalmic form into the chain of febrile action; and what I have seen since, in other situations, confirms me in the opinion that it ought to be so placed.

*Cure.*—Ophthalmia has been so fully described by recent writers, that it is not necessary to make any observations in this place concerning its history. The cure of the recent disease is also so simple, that all I have to say upon it may be comprised in a very few words. It depends principally upon sudden depletion by abstraction of blood. Bleeding is now admitted by all army-surgeons to be the first and the most important remedy. The introduction of it into the British army, to the extent now practised, is due, if I am not mistaken, to Dr. Borland, inspector of hospitals. The ophthalmia had been a troublesome disease among the British military ever since the return of the troops from Egypt; but it prevailed to such extent in the year 1805 as to occasion considerable alarm; for, if it did not destroy life, it destroyed sight, which is tantamount to the life of a soldier. It appeared with violence among some corps which were quartered on the coast of Kent, particularly at Shornecliffe. Dr. Borland, who was then assistant to the inspector-general of hospitals, repaired to Shornecliffe, I believe as a volunteer, to satisfy himself by actual inspection. After inspection, he proceeded to act; and, from a train of well-considered experiments, digested a plan of treatment through which it was safely and easily cured. When the efficacy of the plan was satisfactorily established, instructions were sent from the Medical Board Office, under sanction of a member of the board, —viz. Inspector-general Knight,—detailing the manner of proceeding, and recommending the execution of it to the attention of regimental surgeons throughout the line. Where the plan recommended, the basis of which consists in bleeding, is applied in time and carried to the proper extent in application, it rarely fails in arresting the disease. On this subject I do not know that there is any difference of opinion, in so far as respects recent ophthalmia: the chronic does not yield to the same treatment; but the chronic is not here under view,—it is not known where the means now recommended have been applied with skill and decision during the early stage.

The ophthalmic form of fever prevails among the troops in the West-Indies, in some situations and in some corps more than others. It was frequent among the soldiers of the 90th regiment, one of the regiments which served in the expedition to Egypt in the year 1803. The plan of cure pursued in this corps was depletion, but the depletion was not always sufficiently prompt and sufficiently extensive: hence the cure was often tedious and not always perfect;—the sight of several was blemished, and few were dismissed from the hospital in less than six weeks.

The ophthalmic form of fever did not often fall under my own observation in its recent stages, during the period to which this sketch relates; but it fell under it often enough to prove to me that the cure was not a matter of difficulty, if the plan was well laid, and if the suitable means were applied before the structure of the eye was actually violated. Abstraction of blood is the first remedy, and the remedy of principal dependence; the one, in fact, without which others are of very uncertain effect. The quantity necessary to assure the arrest of a disease of violence cannot be estimated at less than from three to four pounds; but, whatever the amount may be, the effect must be assured before the arm is bound up,—that is, remission from pain, and disappearance of red and turgid veins. The disappearance of the red veins indicates that the point is attained, and determines the amount of the measure. When the course of the disease has been arrested by the abstraction of blood, emetics, purgatives, diaphoretics, diluents, blisters to the temples and nape of the neck, equal and well-adjusted pressure upon the ball of the eye by compresses wet with camphorated eye-water, or acetated water of ammonia, during the abstraction of blood, and continued under the operation of all the other evacuating processes, are the principal means which assist in the removal of the symptoms, and which prevent their recurrence.

An extreme degree of irritability,—a degree so great that the patient cannot open his eye or bear the slightest impression of light,—often remains for some time after the appearances of actual inflammation are removed. In a case of this kind, an experiment was made with infusion of the herb euphrasia, or eye-bright. The subject, an European serjeant in the 8th West-India regiment, a corps of Africans, had been bled largely, taken emetics and purgatives, and was blistered at the neck and on the temples. The redness of the ball of the eye was removed, but irritability remained to such an extreme degree that he could not bear the slightest impression of light without intolerable pain. A draught of strong infusion of eye-bright, in quantity about half a pint, was given while he was in this condition. He recovered the power of opening his eyes by his own will instantaneously, even while in the act of swallowing; and he, in fact, recovered it to such extent as enabled him to look steadily into sunshine. When the irritability returned, or threatened to return, it was removed by a repetition of the draught.—The herb eye-bright may, therefore, I think, from this and other similar experiments, be considered as a valuable aid in the cure of ophthalmia. By help of it, after the inflammatory state is removed by bleeding, and prevented from returning by the judicious employment of emetics, purgatives, blisters, &c. the disease may, if I can venture to speak from limited experience, be perfectly cured in five or six days; while, under feeble and temporizing treatment, it often continues for as many weeks, and even sometimes for as many months.—*Sketches of Febrile Diseases.*

The mention of Euphrasia reminds us of that disposition, which appears to us much too prevalent, of reducing medicine to the same simplicity as the other arts or sciences. In our opinion, it does honour to Dr. Jackson, that he is not afraid of the obloquy which may attend the revival of such remedies as cobweb and euphrasia. What are most of our powerful remedies, but those of which the ancients were afraid, or which have at different times been discarded? When we consider the complicated nature of the human frame, we should rather feel surprised at the uniformity with which many remedies operate, than at peculiar idiosyncrasies, or even peculiar epidemics, under which it may be necessary to vary our practice. Of this we endeavour to remind our readers as often as we complete a volume.—*Et quoniam, &c.*—Edit.

